# Long-Term Electrocardiographic Changes in Healthcare Workers Following Mild to Moderate Cases of Coronavirus (COVID-19): A Longitudinal Observational Study

**DOI:** 10.3390/healthcare13151799

**Published:** 2025-07-24

**Authors:** Luca Coppeta, Giuseppina Somma, Stella Andreadi, Andrea Attanasio, Andrea Magrini, Cristiana Ferrari

**Affiliations:** 1 Department of Biomedicine and Prevention, University of Rome Tor Vergata, 00133 Rome, Italy; luca.coppeta@uniroma2.it (L.C.); giuseppina.somma@ptvonline.it (G.S.); stella.andreadi@ptvonline.it (S.A.); andrea.magrini@uniroma2.it (A.M.); 2 PhD Program in Social, Occupational and Medico-Legal Sciences, Department of Biomedicine and Prevention, University of Rome Tor Vergata, 00133 Rome, Italy

**Keywords:** COVID-19, electrocardiographic changes, healthcare workers, SARS-CoV-2, electrocardiogram, heart rate, PR interval, autonomic dysfunction

## Abstract

**Background**: The cardiovascular effects of SARS-CoV-2, including autonomic dysregulation, are becoming increasingly recognized, even following mild infections. However, long-term electrocardiographic (ECG) changes remain poorly characterized. **Methods**: We conducted a prospective study of 151 unvaccinated healthcare workers with RT-PCR-confirmed mild to moderate SARS-CoV-2 infection. Standard 12-lead ECGs were recorded before infection (T0) and at 6–12 months (T1) and >12 months (T2) after infection. Key parameters included heart rate (HR), PR interval, QRS duration, and corrected QT interval (QTc). **Results**: Heart rate (HR) increased transiently at T1 (*p* < 0.05) and normalized by T2. Mild but persistent PR interval shortening was observed at both follow-ups (*p* < 0.01). There were no significant changes in QRS or QTc intervals. No arrhythmias or conduction blocks occurred. ECG alterations were not associated with sex or age, except for greater PR shortening in males. **Conclusions**: Mild SARS-CoV-2 infection can result in transient sinus tachycardia and subtle PR shortening, which is likely to be a post-viral autonomic effect. Long-term ECG surveillance appears unnecessary in asymptomatic cases.

## 1. Introduction

Since the onset of the pandemic, an increasing body of evidence has demonstrated that the virus affects more than just respiratory symptoms, particularly impacting the cardiovascular system. Of particular concern are the most common sequelae, which include persistent changes in heart rhythm. While severe cardiac complications such as myocarditis, arrhythmia, and thrombotic events have been well documented in hospitalized patients, there is growing evidence to suggest that individuals not in hospital may also experience subtle and persistent cardiac changes during the post-acute and long-term phases of the disease (e.g., palpitations, chest discomfort, intolerance to exercise, and inappropriate sinus tachycardia). These have often been reported in individuals with post-acute sequelae of SARS-CoV-2 infection (PASC), also known as long COVID. Initial symptoms may include conditions such as inappropriate sinus tachycardia, postural orthostatic tachycardia syndrome (POTS), and, though less frequently, new-onset atrial fibrillation or other arrhythmias [1,2,3,4]. The pathophysiological mechanisms underlying these cardiac rhythm disturbances have not yet been fully clarified. However, potential contributing factors have been proposed, including autonomic nervous system dysfunction, myocardial inflammation, and persistent viral reservoirs or immune activation [5,6,7,8]. Although arrhythmic events are generally considered non-life-threatening, their chronic nature has been shown to significantly impact quality of life and functional capacity [9,10,11]. Despite rhythm disturbances being recognized early in long-COVID patients, there is a lack of longitudinal data assessing their long-term trajectory [4,7,12]. It is essential to determine whether these initial alterations persist, improve, or resolve over time to guide prognosis and management strategies.

The present study focuses on standard electrocardiographic markers, including heart rate, PR interval, QRS duration, and QTc interval, which are measured over a period of 6–12 and >12 months following infection. The objective of the present study was to track the same individuals across time points in order to capture low-grade or reversible changes that may be undetected in cross-sectional analyses. This approach was taken with a view to improving understanding of the cardiovascular recovery trajectory in low-risk populations presenting with early post-COVID-19 cardiac autonomic symptoms. Specifically, we will assess whether these alterations regress during a follow-up period of more than 12 months after the acute infection.

## 2. Materials and Methods

This longitudinal observational study enrolled 151 healthy healthcare workers diagnosed with mild to moderate SARS-CoV-2 infections between March and December 2020, before the availability of vaccination programs. Diagnosis was confirmed through reverse transcriptase–polymerase chain reaction (RT-PCR) testing for all individuals.

All participants had received a standard 12-lead electrocardiogram (ECG) within the previous six months as part of their routine annual occupational health screening. The inclusion criteria included an age range of 18 to 65 years, an absence of pre-existing cardiovascular disease, and the availability of baseline and follow-up ECGs. The age cap of 65 years was set to minimize the impact of aging-related factors and ensure a more consistent study population. While this improves the study’s internal validity, it limits its generalizability to older adults, who may respond differently to the effects of the virus and the subsequent period. Including older cohorts in future research is essential in order to validate findings and inform age-inclusive care strategies. Each subject underwent a follow-up ECG within six months of recovering from SARS-CoV-2 infection (early post-infection phase) and again after two to three years (late post-infection phase). All ECGs were recorded using standard equipment and procedures and were subsequently interpreted independently by two experienced cardiologists who were blinded to the clinical information and to the time points.

The following electrocardiographic (ECG) parameters were assessed and classified for each participant. Heart rate (HR) was measured in beats per minute (bpm) and categorized as bradycardia if it was below 60 bpm, normal if between 60 and 100 bpm, or tachycardia if it was above 100 bpm. The PR interval was measured in milliseconds (ms) and classified as normal (120–200 ms), shortened (<120 ms), or prolonged (>200 ms). The QT interval was corrected for heart rate using Bazett’s formula (QTc) and categorized as normal, borderline, or prolonged based on sex-specific thresholds (e.g., QTc > 450 ms in males and >470 ms in females was considered prolonged). QRS duration was measured in milliseconds and classified as normal if <120 ms or prolonged if ≥120 ms. Morphology was analyzed when applicable, for example, in cases of bundle branch blocks. All measurements were obtained from resting 12-lead ECGs and reviewed by trained personnel using standardized protocols.

Electrocardiographic parameters, heart rate (HR), PR interval, QRS duration, and corrected QT interval (QTc), were measured at three time points: T0 (baseline): before SARS-CoV-2 infection; T1 (follow-up): 6 to 12 months after infection; and T2 (late follow-up): more than 12 months after infection.

Demographic and clinical data, including age, sex, body mass index, and comorbidities, were collected at baseline. Quantitative ECG parameters were reported as the mean ± standard deviation (SD) or the median with interquartile range (IQR), depending on the normality of the distribution. Comparisons across the three time points (pre-infection, early post-infection, and late post-infection) were conducted using repeated-measures ANOVA for normally distributed variables or Friedman tests for non-parametric data. Paired categorical variables were analyzed using McNemar’s test or Cochran’s Q test, as appropriate.

All statistical analyses were conducted using SPSS software version 25.0, with a significance threshold established at *p* < 0.05.

## 3. Results

A total of 151 adult subjects, with a mean age of 44.6 years (±9.4), was included in the study. The cohort consisted of 96 females (63.6%) and 55 males (36.4%). Electrocardiographic parameters were assessed at three distinct time points: baseline (T0, before SARS-CoV-2 infection), follow-up (T1, 6–12 months post-infection), and late follow-up (T2, over 12 months post-infection).

The baseline heart rate was normal in 126 individuals. Among those with abnormalities, 23 patients exhibited bradycardia (heart rate <60 beats per minute), and two presented with tachycardia (heart rate >100 beats per minute). Regarding atrioventricular conduction, the baseline PR interval was within the normal range (120–200 ms) for 143 individuals. Six individuals had a short PR interval (<120 ms), and two were diagnosed with a first-degree atrioventricular (AV) block (>200 ms). The baseline QRS duration was normal (70–110 ms) in 147 individuals, while four individuals exhibited a prolonged QRS duration (>110 ms), which suggests a possible intraventricular conduction delay. A normal QTc interval (330–440 ms in males and 330–460 ms in females) was observed in 144 patients, accounting for 95.4% of the cohort. A short QTc interval (<330 ms), indicating accelerated repolarization, was present in four patients (2.6%). A prolonged QTc interval (above the gender-specific upper limit) was identified in three patients (2.0%). At baseline, a normal axis (between −30° and +100°) was observed in 147 patients (97.4%), while an abnormal axis was present in four patients (2.6%). Comparative data of ECG parameters at baseline, T1, and T2 are shown in Table 1.

The heart rate was 69.9 ± 12.3 bpm at baseline and increased to 71.6 ± 11.5 bpm at T1. According to the paired t-test, this change was statistically significant (*p* < 0.05). At T2, the heart rate had returned to 68.5 ± 11.8 bpm, and the difference from the baseline reading was not statistically significant (*p* = 0.091). This pattern suggests a transient elevation in heart rate in the months following a SARS-CoV-2 infection. The PR interval decreased significantly over time. At baseline, the mean PR interval was 158.6 ± 23.3 ms, dropping to 152.7 ± 21.7 ms at T1 and to 153.4 ± 20.4 ms at T2. The difference between T0 and T1 was significant (*p* < 0.01), as was the change from T0 to T2 (*p* = 0.0021), despite the distributions at T0 and T2 not being normal. These findings indicate a persistent reduction in atrioventricular conduction time following infection. QRS duration remained stable throughout the observation period, with mean values of 91.0 ± 8.2 ms at T0, 91.1 ± 9.4 ms at T1, and 91.2 ± 9.1 ms at T2. No statistically significant changes were observed between T0 and T1 (*p* = 0.8729) or between T0 and T2 (*p* = 0.7978). Regarding QT, a mild but statistically significant increase was observed between T0 and T2 (from 380.9 ± 32.4 ms to 384.8 ± 27.6 ms; *p* = 0.0348). The difference between T0 and T1 was not statistically significant (*p* = 0.2708).

At T2, a mild but statistically significant increase in QTc interval was observed. Despite achieving statistical significance, this variation remained within normal physiological ranges and does not appear to have clinical relevance in a generally healthy population. However, it is possible that these results reflect transient, benign adaptations during the post-acute recovery phase.

As shown in Table 2, subsequent analyses examined the main electrocardiographic changes between baseline (T0) and the final follow-up (T2) in male and female participants separately, using paired *t*-tests. In the male group, a statistically significant reduction in heart rate was observed from T0 to T2, with a mean difference of −3.02 bpm (*p* < 0.05). A significant shortening of the PR interval was also noted within this timeframe, with a mean reduction of −9.80 ms (*p* < 0.01). No significant variation was found in QRS duration (−0.84 ms, n.s.). Although the QTc interval showed a slight mean increase of 1.62 ms, this change was not statistically significant (*p* = n.s.). Among female participants, no ECG parameter showed a statistically significant change from baseline to late follow-up. Heart rate remained essentially stable, with a mean change of −0.49 bpm (*p* = 0.51), and the PR interval showed a modest, non-significant reduction of −2.56 ms *(p* = 0.51). Likewise, the minor variations observed in QRS duration (+0.72 ms, n.s.) and QTc interval (+2.47 ms, n.s.) were not statistically significant.

**Figure 1:**
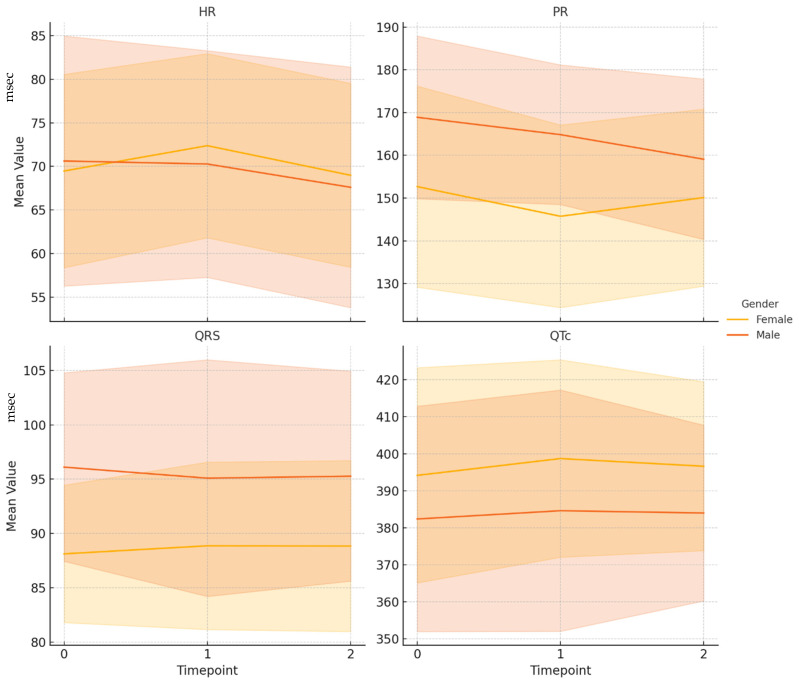
Trend of ECG parameters over time by gender.

Main findings are shown in Figure 1.

We assessed the correlation between variations in heart rate (HR), PR interval, QRS complex, and QT interval corrected (QTc) values (from baseline T0) and the number of days between T0 and subsequent evaluations (T1 and T2) to determine whether the time elapsed since SARS-CoV-2 infection influenced changes in electrocardiographic parameters. No significant association was found between changes in heart rate and time at either T1 (ρ = −0.018, *p* = 0.8305) or T2 (ρ = 0.118, *p* = 0.1501). This indicates negligible effect sizes and no clinically relevant trends. Similarly, variations in QRS duration and QTc interval over time were not statistically or clinically significant. However, a weak but statistically significant inverse correlation was observed between PR interval variation and the number of days to T2 (ρ = −0.171, *p* = 0.0356). While the effect size is small, this may suggest a minor time-dependent improvement in atrioventricular conduction. However, given the low magnitude of the correlation, its clinical relevance remains uncertain and requires cautious interpretation.

Finally, logistic regression was performed to verify whether the reduction in heart rate (HR) within the group was related to sex and age. The onset of bradycardia was used as the dependent variable, and male sex and age greater than 45 years were used as the independent variables. Neither variable was significantly associated with the onset of bradycardia; however, older age approached statistical significance (*p* = 0.064).

## 4. Discussion

This longitudinal study involved 151 healthcare workers who were recovering from mild to moderate SARS-CoV-2 infection. We documented transient increases in resting heart rate and a subtle, yet statistically significant, shortening of the PR interval over time. No significant changes were observed in QRS duration or the corrected QT (QTc) interval. This is consistent with previous reports suggesting that structural conduction disturbances are uncommon in non-hospitalized cases [13]. Although the observed PR interval reduction was modest in magnitude (ρ = −0.171, *p* = 0.0356), it may reflect subtle alterations in atrioventricular nodal conduction. These alterations could be mediated by autonomic nervous system dysregulation or transient inflammatory effects on cardiac conduction pathways. While the effect size suggests limited clinical significance in an otherwise healthy cohort, the consistent directionality of the change raises the possibility of time-dependent physiological adaptation or subclinical autonomic remodeling during post-acute recovery.

From a clinical perspective, routine ECG monitoring for PR interval shortening is not currently indicated in asymptomatic individuals, particularly in the absence of bradyarrhythmias, symptoms of syncope, or baseline conduction disorders. However, closer follow-up could be considered in specific populations, such as older adults, patients with known conduction system disease, or individuals exhibiting persistent cardiovascular symptoms. Furthermore, as SARS-CoV-2 has been shown to affect autonomic regulation and cardiac electrophysiology, incorporating autonomic function testing and long-term rhythm monitoring into future studies could clarify whether such PR changes have any prognostic or pathophysiological relevance.

In summary, while PR shortening in this cohort appears to be a mild, likely reversible phenomenon with uncertain clinical implications, it could be useful for signaling future investigations into post-viral cardiac autonomic recovery.

While the underlying mechanism was not investigated in this observational study, we can propose some explanatory hypotheses to account for these changes. The increase in heart rate in the early stages of post-infection likely reflects transient autonomic dysregulation or deconditioning following SARS-CoV-2 infection. Many patients with post-acute sequelae of SARS-CoV-2 infection (long COVID) experience inappropriate sinus tachycardia in the months following infection. Our data show that this effect, while notable at 6–12 months post-infection, tended to resolve by the time of the later follow-up (>12 months), with the average heart rate returning to near baseline levels [2,5,9]. This trajectory suggests that, for individuals with mild initial illness, SARS-CoV-2-related sinus tachycardia is self-limited and improves over time, mirroring the recovery of autonomic balance. In male participants, a statistically significant reduction in heart rate was observed more than one year after baseline [14,15]. Although statistically significant, the observed PR interval shortening was modest (~5–10 ms) and remained within normal physiological limits. Consequently, its clinical relevance appears to be limited. These subtle variations may be indicative of transient autonomic changes, which could be influenced by confounding factors such as stress and lifestyle alterations that were prevalent among healthcare workers during the pandemic. The absence of a similar trend in females suggests the possible interplay of sex-specific or behavioral factors.

In contrast, the shortening of the PR interval observed at the initial time point was still evident at the final follow-up. Although the absolute change was small, its persistence may indicate a lasting alteration in atrioventricular nodal conduction [13]. This mechanism was not investigated in the present study and remains speculative. One plausible explanation is an imbalance in the autonomic nervous system: reduced vagal tone or increased sympathetic tone post-infection could shorten AV nodal conduction time [5,16]. This finding aligns with the growing evidence suggesting that autonomic dysfunction can persist following a SARS-CoV-2 infection. Notably, a published study documented postural orthostatic tachycardia syndrome (POTS) and related autonomic disorders developing after SARS-CoV-2 infection, with 85% of patients still reporting autonomic symptoms (e.g., tachycardia and palpitations) six to eight months later [17]. Although our cohort was generally healthy and did not undergo formal autonomic testing, the persistent PR shortening may be a subtle ECG indicator of post-COVID-19 autonomic changes. Importantly, the magnitude of PR shortening observed is unlikely to result in clinical heart block or other conduction pathology. Instead, it may reflect benign variations in autonomic tone [13]. We found no evidence of progressive PR prolongation or high-grade atrioventricular (AV) block in any subject, which is reassuring. Similarly, the absence of significant changes in the QRS or QTc intervals over more than 12 months suggests that the electrophysiological properties of the myocardium remain largely intact following a mild case of SARS-CoV-2 infection [18,19]. In the event of substantial myocardial scarring or repolarization abnormalities (as can occur in myocarditis), QRS prolongation, pathological Q waves, or QTc changes would be expected. However, none of these were observed in the present study.

The present results build upon and provide context for previous observations of long-term cardiac rhythm disturbances. A substantial number of population-based studies have reported an increased incidence of arrhythmias following a positive diagnosis of SARS-CoV-2, the novel coronavirus responsible for severe acute respiratory syndrome coronavirus 2 (SARS-CoV-2).

Compared to the matched controls, survivors of the SARS-CoV-2 virus had a significantly higher 12-month risk of a broad range of dysrhythmias, including atrial fibrillation, atrial flutter, sinus tachycardia, and others. This excess risk was evident even among those with mild, non-hospitalized cases, although it was most pronounced in hospitalized patients or those who required intensive care [2,20]. The findings of this study are consistent with epidemiological data, which has documented sinus tachycardia as part of the post-COVID-19 syndrome [9,16]. However, no serious arrhythmias, such as atrial fibrillation or ventricular arrhythmias, were detected in the cohort under investigation. This is likely a consequence of the relatively small sample size and the young, healthy nature of the studied population. The incidence of such arrhythmias in mild survivors of SARS-CoV-2 infection is low (e.g., the incidence of atrial fibrillation is around 30 per 1000 person-years in SARS-CoV-2 cases versus 5 per 1000 in controls), so a cohort of 151 individuals may simply be too small to detect these rare events [2,20,21]. Similarly, Pranata et al. (2022) and Garcia-Zamora et al. (2021) conducted a systematic review and meta-analysis of cardiac arrhythmias in patients with SARS-CoV-2 infection and found that approximately 10% of hospitalized patients experience arrhythmia during the acute phase of infection, with atrial arrhythmia being the most common type [21,22]. Together with our findings, this highlights that while mild cases of SARS-CoV-2 infection tend to manifest as transient sinus tachycardia, more severe cases can precipitate cardiac rhythm disturbances.

Considering the early concerns about myocardial injury associated with SARS-CoV-2, our observation of minimal long-term changes in ECG intervals is noteworthy. Puntmann et al. (2020) reported unexpectedly high rates of cardiac involvement on MRI scans in patients who had recently recovered from mild cases of the virus, with 78% exhibiting some abnormality and 60% meeting the criteria for ongoing myocardial inflammation [23]. These findings raised concerns about subclinical myocarditis and potential arrhythmic complications, even in individuals who appeared to be healthy [23,24]. In contrast, subsequent studies (including our own) have provided more reassuring results for this population [25,26]. We found no ECG evidence of latent myocardial damage, such as bundle branch block, significant ST-T changes, or arrhythmogenic QTc prolongation, in our participants up to 1 year post-infection. Our results are consistent with the growing consensus that, although acute cardiac injury can occur in patients with SARS-CoV-2 infection, the majority of individuals with mild initial illness do not develop clinically significant structural heart disease or arrhythmias in the long term [13]. Advanced imaging techniques, such as cardiac MRI, may still detect subtle abnormalities in some of our subjects. However, the absence of ECG changes and the benign clinical course observed provide reassurance that these changes, if present at all, are not resulting in electrical instability or functional impairment [13,25].

The findings of our study present several important clinical implications. Firstly, they underline that transient heart rate elevation is a common feature during the post-acute phase of mild cases of SARS-CoV-2 infection, but this typically improves over time for most patients [9,16]. Healthcare providers should recognize that this finding in the months following recovery from mild cases of the disease can be part of the convalescent process. This offers an encouraging message for patients experiencing palpitations or tachycardia as part of the long-term effects of the virus: these symptoms are generally self-limiting. However, careful follow-up is still essential, as a small number of patients may develop persistent autonomic dysfunction (such as POTS), which could be addressed through targeted therapy [17,27]. Our observation of PR interval shortening, potentially a marker of sympathetic predominance, even at late follow-up, indicates that some degree of autonomic imbalance can persist. Therefore, clinicians should consider monitoring patients for orthostatic symptoms or conducting autonomic function tests in patients with ongoing complaints, even if the standard ECG appears normal [5,14].

Despite the fact that the present study did not directly evaluate autonomic nervous system function through dedicated tools (e.g., heart rate variability analysis or autonomic testing), it is important to note that previous research has highlighted the significance of clinical context in shaping post-infectious autonomic responses. In particular, factors such as disease severity, baseline fitness level, comorbidities, and the presence or absence of long-term symptoms appear to modulate autonomic outcomes more significantly than the infection itself. For instance, recent studies have demonstrated that autonomic dysfunction is more commonly detected in patients with persistent post-COVID-19 symptoms or in those with pre-existing vulnerabilities [28,29]. These findings lend support to the interpretation that the subtle ECG changes observed in our otherwise healthy cohort recovering from mild illness are likely to be benign and context-dependent.

Secondly, the absence of pathological ECG changes in our cohort is reassuring regarding long-term cardiac outcomes in cases of mild COVID-19 [13,25]. This suggests that routine ECG follow-up beyond the first year may not be necessary for asymptomatic individuals with a history of mild disease, provided the initial post-recovery ECG is normal [26,30]. Our findings do not support the need for extensive cardiac screening (e.g., serial ECGs or echocardiograms) for all individuals with mild SARS-CoV-2 infection. Instead, a symptom-driven approach to cardiac evaluation is reasonable. Patients presenting with chest pain, unexplained exertional dyspnea, or palpitations should, of course, be evaluated for myocarditis or arrhythmias. However, in the absence of such red flags, long-term cardiac monitoring of otherwise healthy post-COVID-19 patients is likely to have limited value [27]. This aligns with the clinical observation that most mild cases resolve without incident and that serious cardiac complications tend to occur in individuals with severe acute illness or pre-existing cardiovascular conditions [20]. For occupational health and cardiology practices, this means that while interim checks (e.g., at six months) might detect issues such as sinus tachycardia, these abnormalities often resolve within 12 months, enabling most individuals to resume their normal activity levels without experiencing any long-term cardiac impairment [9].

We acknowledge the limitations of our study. First, our cohort consisted solely of healthcare workers, who may not be representative of the general population. Since these individuals were relatively young and without severe comorbidities, our findings may not apply to older patients or those with significant pre-existing cardiovascular risk factors. Second, we did not systematically collect data on physical activity, fitness, or other lifestyle factors during the follow-up period. Changes in exercise habits or body weight could influence resting heart rate and ECG parameters over the long term. For instance, post-COVID-19 deconditioning may result in higher heart rates, whereas improved fitness could lead to lower heart rates and a slightly shorter PR interval due to enhanced vagal tone. Without detailed lifestyle questionnaires or activity monitoring, it is not possible to definitively attribute the observed change in heart rate and PR to the direct effects of COVID-19 rather than secondary factors. Furthermore, no formal autonomic function tests, such as tilt-table tests or heart rate variability analysis, were performed. Such tests could have strengthened our interpretation of PR shortening as an autonomic phenomenon. Without direct measures of autonomic output or inflammatory markers, the mechanistic underpinnings of the ECG changes remain speculative.

Another limitation is that our ECG assessments were restricted to standard 12-lead recordings taken while at rest at three different time points. We did not utilize continuous ambulatory monitoring (e.g., Holter monitors) or implantable loop recorders. This means that transient or asymptomatic arrhythmias, such as paroxysmal atrial fibrillation or isolated ectopic beats, may have remained undetected. Consequently, the true burden of subclinical rhythm disturbances in the post-acute phase may be underestimated.

Additionally, while we carefully excluded individuals with known cardiac disease and confirmed that none of the subjects were taking beta-blockers or other rate-modulating medications during the follow-up period, we cannot rule out the potential influence of other medications or supplements that participants took independently. It is important to note that no beta-blockers were used, confirming that the observed heart rate trends were unaffected by pharmacological rate control. At the same time, this suggests that participants largely did not require medical therapy for palpitations, indicating that their symptoms were manageable. Lastly, given the observational nature of the study, we cannot determine whether SARS-CoV-2 infection caused the PR interval shortening; we can only conclude that the change occurred following the infection.

The last limitation of this study is the absence of a control group of individuals who have not been infected. Although within-subject designs, in which individuals are evaluated over time, offer valuable insights into changes associated with SARS-CoV-2 infection, they do not fully account for normal variability that can occur independently of the disease. Aging, psychological stress, evolving lifestyle habits, and fluctuations in physical activity levels are all factors that can influence physiological and cognitive outcomes over time. Without a control group of uninfected individuals assessed under similar conditions, it is difficult to determine whether the observed changes are directly attributable to the virus or part of broader population-level trends. For instance, during the pandemic, both infected and uninfected individuals experienced disruptions to their daily routines, increased sedentary behavior, and heightened psychological stress, each of which could affect long-term health outcomes. A matched control cohort, ideally stratified by age, sex, socioeconomic status, and baseline health, would enable more rigorous discrimination between disease-specific effects and general temporal variability. This approach would strengthen causal inferences and enhance the interpretability and external validity of study findings.

On the other hand, the primary strengths of our study are its longitudinal design, where each subject serves as their own control (comparing baseline and post-infection ECGs), and the inclusion of subjects who have never been vaccinated. Indeed, vaccination has been shown to protect against the symptoms and complications of the infection, including cardiovascular effects [31,32].

## 5. Conclusions

In summary, our longitudinal analysis of ECG changes in healthcare workers recovering from mild to moderate COVID-19 revealed a transient increase in sinus heart rate and a mild but persistent shortening of the PR interval. There were no lasting changes in ventricular conduction or repolarization intervals. These results suggest that long-term cardiac structural sequelae are unlikely in this group, despite the observed transient autonomic effects. The results align with and add nuance to the growing body of literature on cardiac manifestations of the long-term effects of the virus, confirming that tachycardia and autonomic disturbances are common in the early stages of recovery, while also providing reassurance that these effects typically improve over time. Ongoing vigilance in the post-COVID-19 period is warranted to identify the minority of patients who may develop sustained arrhythmias or autonomic dysfunction. However, in the absence of symptoms or pre-existing cardiac conditions, routine long-term cardiac surveillance does not appear to be necessary for individuals recovering from mild SARS-CoV-2 infection, as the observed changes, such as PR interval shortening, are subtle, non-progressive, and unlikely to carry clinical consequences. Future studies incorporating comprehensive autonomic testing, biochemical markers, and imaging could further elucidate the mechanisms behind post-COVID-19 ECG changes and definitively determine whether subtle cardiac involvement, such as that seen on MRI scans in some cases (pubmed.ncbi.nlm.nih.gov), has any long-term clinical significance. Overall, our findings contribute to a more optimistic outlook for cardiovascular recovery after mild cases of the virus, emphasizing that, for most individuals, the heart’s electrical function remains resilient in the face of this novel infection.

## Figures and Tables

**Table 1:** Comparative data of ECG parameters at baseline, T1, and T2 time points.

Parameter	Category	Baseline ECG n (%)	ECG at T1 n (%)	ECG at T2 n (%)	Baseline/T1 *p*-Value	Baseline/T2 *p*-Value
Heart Rate (HR)	normal	126 (83.4%)	133 (88.1%)	117 (77.5%)	<0.01	<0.01
	bradycardia	23 (15.2%)	16 (10.6%)	32 (21.2%)		
	tachycardia	2 (1.3%)	2 (1.3%)	2 (1.3%)		
PR Interval	prolonged	2 (1.3%)	0 (0.0%)	0 (0.0%)	<0.01	<0.01
	normal	143 (94.7%)	143 (94.7%)	143 (94.7%)		
	short	6 (4.0%)	8 (5.3%)	8 (5.3%)		
QRS Duration	prolonged	2 (1.3%)	2 (1.3%)	2 (1.3%)	n.s.	n.s.
	normal	149 (98.7%)	149 (98.7%)	149 (98.7%)		
QTc Interval	prolonged	3 (2%)	4 (2.6%)	1 (0.7%)	<0.01	<0.01
	normal	144 (95%)	144 (95.4)	148 (98.0)		
	short	4 (2.6%)	3 (2%)	2 (1.3%)		
QRS Axis	normal	147 (97.4%)	151 (100%)	149 (98.7%)	<0.01	<0.01
	abnormal *	4 (2.6%)	0 (0%)	2 (1.3%)		
Total (N)		151	151	151		

* Abnormal QRS axis refers to left or right axis deviation outside normal physiological range. n.s.: not significant.

**Table 2:** Electrocardiographic parameters by gender over time at baseline, T1, and T2 time points.

Parameter	Baseline ECG (Mean ± SD)		ECG at T1 (Mean ± SD)		ECG at T2 (Mean ± SD)	
	Female	Male	Female	Male	Female	Male
Heart Rate (HR)	69.47 ± 11.09	70.62 ± 14.35	72.38 ± 10.57	70.27 ± 13.01	68.98 ± 10.55	67.6 ± 13.8
PR Interval	152.69 ± 23.52	168.89 ± 19.02	145.75 ± 21.35	164.84 ± 16.32	150.12 ± 20.7	159.09 ± 18.76
QRS Duration	88.12 ± 6.32	96.11 ± 8.66	88.86 ± 7.71	95.09 ± 10.88	88.84 ± 7.87	95.27 ± 9.65
QTc Interval	385.88 ± 32.21	372.22 ± 31.21	387.44 ± 28.02	376.76 ± 37.45	389.39 ± 26.0	376.87 ± 28.79

## Data Availability

The data supporting the reported results can be made available upon request to the authors.
